# Evaluation of the Impact of the Plastic BioSand Filter on Health and Drinking Water Quality in Rural Tamale, Ghana

**DOI:** 10.3390/ijerph9113806

**Published:** 2012-10-24

**Authors:** Christine E. Stauber, Byron Kominek, Kaida R. Liang, Mumuni K. Osman, Mark D. Sobsey

**Affiliations:** 1 Institute of Public Health, Georgia State University, P.O. Box 3995, Atlanta, GA 30302, USA; 2 Department of Environmental Sciences and Engineering, Gillings School of Global Public Health, University of North Carolina-Chapel Hill, Campus Box 7431, Chapel Hill, NC, 27599-7431, USA; Email: byron.kominek@gmail.com (B.K.); kliang@email.unc.edu (K.R.L.); mark_sobsey@unc.edu (M.S.); 3 Cowater International Inc., NORST, P.O. Box 1476, Tamale, Ghana; Email: hoiinahii@gmail.com

**Keywords:** water quality, *E. coli*, diarrheal disease, water treatment, water filtration

## Abstract

A randomized controlled trial of the plastic BioSand filter (BSF) was performed in rural communities in Tamale (Ghana) to assess reductions in diarrheal disease and improvements in household drinking water quality. Few studies of household water filters have been performed in this region, where high drinking water turbidity can be a challenge for other household water treatment technologies. During the study, the longitudinal prevalence ratio for diarrhea comparing households that received the plastic BSF to households that did not receive it was 0.40 (95% confidence interval: 0.05, 0.80), suggesting an overall diarrheal disease reduction of 60%. The plastic BSF achieved a geometric mean reduction of 97% and 67% for *E. coli* and turbidity, respectively. These results suggest the plastic BSF significantly improved drinking water quality and reduced diarrheal disease during the short trial in rural Tamale, Ghana. The results are similar to other trials of household drinking water treatment technologies.

## 1. Introduction

Many communities, especially in rural sub-Saharan Africa, still face significant challenges to provide access to improved drinking water sources and are struggling to meet the Millennium Development Goals for water and sanitation [1]. In Ghana, it is estimated that 93% of the urban population and 76% of the rural population have access to improved water and about 17% of urban and less than 10% of rural population have access to improved sanitation [2]. The lack of access to improved water and sanitation contribute significantly to diarrheal disease in the population. Specifically, the Ghana Demographic and Health Survey [2] suggests that diarrheal disease is a significant cause of morbidity and mortality in children less than five years of age, with 1 in 5 having reported diarrheal disease in the two weeks preceding the survey. In the Northern region of Ghana, the two-week prevalence of diarrheal disease was almost 33% [2]. 

An immediate solution to address the lack of access to safe water is household water treatment (HWT), which allows households to treat drinking water at the point of consumption to improve its quality. Studies of HWT have shown that it can reduce the risk of diarrheal disease by 35% or more for a wide range of technologies in different settings and populations [3,4]. One technology that shows promise for areas where water has higher turbidity is the Hydraid® BioSand Water filter (plastic BSF), originally invented by David Manz [5]. This filter, which functions similarly to a slow sand filter, has been modified for intermittent use. The most studied BSFs have been those having concrete housings. The BSF’s advantages are many, including a simple design, durable materials, local fabrication of the concrete housing, and provision of abundant quantities of water [6]. There have been four peer-reviewed published trials [7,8,9,10] examining the health impact of the concrete BSF. These trials suggest that use of the concrete BSF can reduce diarrheal disease by 50% or greater [7,8,9,10]. While the BSF most often implemented is a concrete version, it has a relatively slow rate of daily production, can weigh as much as 500 lbs. when fully installed and can be difficult to transport to remote locations [11].

**Figure 1:**
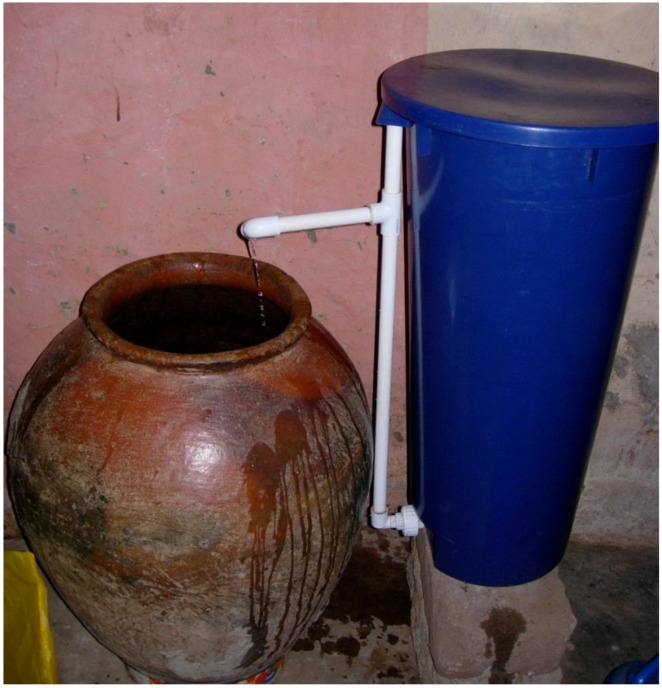
Typical set-up of the plastic BSF in a household in rural Tamale, Ghana (2008).

A version of the BSF having a plastic housing has recently been produced and may overcome the problems in production, distribution and transport of the concrete filter. The plastic BSF is light in weight, stackable and can be produced rapidly in large quantities by injection molding. The plastic version of the BSF tested in this study is licensed by Manz, manufactured by Cascade Engineering and has specific depth and design parameters [5]. Its sand filter bed has a smaller surface area than that of the concrete filter and it has tapered rather than straight side walls (see Figure 1). Furthermore, there is limited evidence of how well it will work in the field to improve water quality and reduce diarrheal disease risks, especially for waters with high turbidity. 

To address the lack of field evidence of the performance of the plastic BSF to improve drinking water quality and reduce diarrheal disease in turbid surface water used by a population with a high diarrheal disease burden, a cluster randomized controlled trial (RCT) was performed in rural communities located in the Northern Region, Tamale, Ghana. This study is one of three RCTs simultaneously performed on the plastic BSF in different geographic regions (including Cambodia [12] and Honduras [13]). This is the first trial of the plastic BSF in this region of the world and only the second BSF RCT in Sub-Saharan Africa [8]. Unique to this region and due to water scarcity, the communities often rely on water stored in “dugouts”, which are shallow surface water impoundments, [14]. The purpose of the RCT was to document the ability of the plastic BSF to improve water quality for both fecal indicator bacteria and turbidity and to reduce diarrheal disease in a setting where the population relies heavily on contaminated, highly turbid surface water for drinking and where the lack of access to water and sanitation are likely to be contributing significantly to morbidity and mortality in children under five years of age [2,14]. 

## 2. Materials and Methods

### 2.1. Research Setting, Study Population, and Participant Recruitment

**Figure 2:**
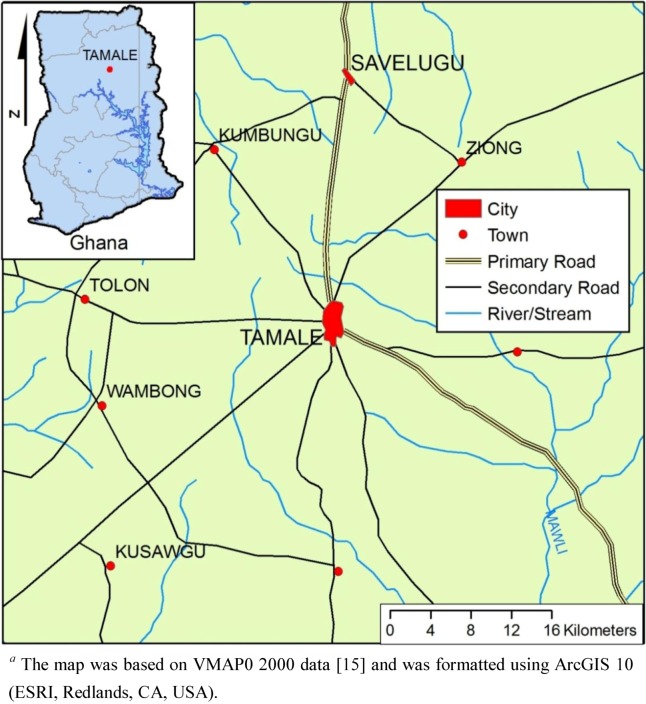
Map *^a^* (1:500,000) indicating the location for the Randomized Controlled Trial of the Plastic Biosand Filter in Tamale, Ghana (2008).

This study (a cluster RCT) of the plastic BSF was conducted in six rural communities in Tamale, Ghana. All villages and the water quality testing laboratory were located within 25 km of the city of Tamale (see Figure 2). Field data collection took place between May to December 2008. The six study communities and their households were selected based on the following criteria: child under the age of five years old, stored drinking water in the home, use of surface water as their primary drinking water source, did not spend most of the day selling goods in Tamale, were within 60 minutes from Tamale during the rainy season, and households agreed to participate. 

Study design and protocols were approved by the Institutional Review Board of the University of North Carolina (IRB #08-0063) and the Ethical Review Committee of the Ghana Health Service. The initial study was powered to detect a 25% reduction in diarrheal disease between the two groups based on an initial prevalence of diarrheal disease of 5%, 80% power and, α = 0.05. We also took into account the clustering of diarrhea within individuals and households and assumed four months of follow up visits as well as four people per household. Based on these sample size parameter values, we estimated the need for approximately 100 households in each study arm.

Prior to recruitment, village elders were approached and informed about the study. If the village elders were interested in participating, individual households were then asked to participate. Households were excluded from the study if they did not have a child less than five years of age and/or did not want to participate. Household recruitment began on 10 May 2008 and was finalized on 16 June 2008, with informed consent obtained during the initial household visit. The purpose of the initial cross-sectional recruitment questionnaire was to collect data on diarrheal disease prevalence in the households, risk factors of diarrheal disease, main drinking water sources, education levels of household members, access to sanitation and presence and type of any drinking water treatment practices. Access to sanitation was assessed via questionnaire and a visual inspection of the facility, if it was present. Lack of access was characterized by the absence of any type of latrine, pour flush toilet or other appropriate sanitation technology. The initial cross-sectional recruitment was completed in six communities and a total of 260 households were recruited. After the initial recruitment, a baseline period of observation was performed prior to intervention with the plastic BSF. The purpose of the baseline data collection period was to characterize and compare diarrheal disease and water quality between what would become the randomly selected intervention (plastic BSF) and control (no plastic BSF) villages.

### 2.2. Intervention

Data collection for the longitudinal portion of the prospective cohort began on 17 June 2008 and was finished on 23 December 2008. Households were visited at one week intervals and asked questions about diarrheal disease and water management practices in the home. During this time period, household water quality was also monitored periodically for total coliforms, *E. coli* and turbidity.

The randomization of villages and installation of plastic BSFs took place during the last week of August and first week of September 2008. Based on discussions with village leaders, randomization at the household level was deemed unacceptable by the majority of the villages. Therefore, the randomization was performed at the village-level. Numbers were assigned to the six villages and three numbers were selected from a random number generator to be the intervention villages. Due to the unequal size of the villages, more households were selected into the control group. At the time of randomization, the six villages ranged in size from 14 to 70 households with children <5 years of age. As a result of randomization, three villages with 70, 58 and 14 households were selected into the control group and three villages with 58, 24 and 33 households were selected into the intervention group (and received the plastic BSF). 

The intervention phase of the study included weekly household observations from September 2008 to December 2008, with a potential of 15 weeks of household observations completed during this time period. All households in the intervention and control groups continued to provide detailed information on weekly levels of diarrheal disease. For household water quality analysis, turbidity was measured every two weeks and bacterial concentrations were measured five times during the intervention period, usually every two weeks.

### 2.3. Diarrheal Disease Surveillance

A consistent system of diarrheal disease surveillance was developed where one person in the household was identified as the primary respondent during the recruitment interview. This person was surveyed weekly about diarrheal disease for all members of the household. Using the following questions “Has anyone in your house had diarrhea in the past one week?” and “If yes, how many times did that person go in one 24-hour period?”, the primary respondents were asked to verbally report any occurrence of diarrhea in the household within the last 7 days. Additional questions on stool appearance (including the appearance of blood), duration of symptoms of diarrhea and use of treatment were also asked. If the case of diarrhea was ongoing at the time of the visit, the case was asked about during the next visit to determine if it had resolved as well as to determine the duration of the case. Overall, there were 12 possible visits prior to plastic BSF installation as the intervention and 15 possible visits after plastic BSF installation, for a total of 27 potential weeks of observation for diarrheal disease surveillance.

### 2.4. Water Quality Sample Collection and Analysis

From the 260 households that were enrolled, samples of drinking water were taken during household visits from both the control and plastic BSF household groups. During the plastic BSF intervention, control households provided a sample of water used for drinking and BSF households providing three water samples at each visit: pre-filtered or untreated water, water directly from the plastic BSF outlet tube, and plastic BSF-treated water that had been stored for drinking. 

Water samples were collected by field staff directly into 500 mL sterile plastic collection bottles. These samples were stored on ice and transported to the World Vision Laboratory at Savelugu, where they were immediately processed (within six hours of collection). All samples were tested for total coliforms and *E. coli* using the IDEXX Colilert® Quantitray 2000® system (IDEXX Laboratories, Westbrook, ME, USA). Most probable numbers (MPN) for total coliforms and *E. coli* were determined using the IDEXX provided MPN table. Turbidity was tested using the Hach 2100P Portable Turbidimeter (Hach Company, Loveland, CO, USA). 

### 2.5. Data Analysis

Data from the initial cross-sectional questionnaire were used to compare the plastic BSF and control groups. Pearson chi-squared tests were used to assess the proportion in each group for the following variables: access to sanitation, main drinking water source, educational attainment, having multiple children <5 years of age in the household, and reported drinking water treatment practices.

The effect of the plastic BSF on diarrheal disease was determined by comparing the longitudinal prevalence of diarrheal disease for all participants in each group, intervention (received BSF) and control (no BSF) using longitudinal prevalence ratios (LPR) generated from Poisson regression. In order to classify a case of diarrheal disease, we used the World Health Organization (WHO) definition of three or more loose or watery stools in at least a 24-hour period. To adjust for clustering within households and the villages, multi-level Poisson regression was performed and all data reported are based on the LPR from the regression model adjusted for clustering. To fit the model, days with diarrheal disease (as counts) and person-days of observation were totaled by individual participant. The multi-level model was used to adjust for clustering because individuals belonged to households which belonged to villages. All diarrheal disease data analysis was performed in Stata 10.0 (Stata, StataCorp, College Station, TX, USA). 

Bacterial concentration and turbidity data were log_10_ transformed and analyzed in Microsoft Excel and Stata 10.0 for graphical presentation and means testing. The bacterial and turbidity reductions achieved by the plastic BSF were calculated as log_10_ reductions: log_10_ reduction = log_10_ influent – log_10_ effluent (Equation 1). Filtered drinking water quality of plastic BSF households was compared both for water taken directly from the filter and for stored filtered water and compared to untreated water from BSF and control households. Paired and unpaired t-tests were used to compare geometric mean log_10_ *E. coli* concentrations and geometric mean turbidities between plastic BSF and control water samples.

## 3. Results

### 3.1. Study Enrollment and Completion

During the initial cross-sectional recruitment interview, six villages and 260 households were recruited into the study. Three villages were later randomized into the BSF intervention group and a total of 117 plastic BSFs were installed in separate households. Three villages were selected to remain as the control villages. All control households were asked to continue normal water management practices for the intervention period. Although this randomization resulted in a small number of clusters, this small number of villages was selected to facilitate weekly follow-up visits. A larger number of villages would have made it logistically challenging to complete all visits within one week (the desired diarrheal disease recall period). Prior to randomization, during the baseline period, nine households (3.4%) dropped out of the study. During the plastic BSF intervention period, a total of seven households (2.3%) dropped out of the study; two households from the control group and five households from the plastic BSF intervention group. 

### 3.2. Baseline Characteristics and Group Comparability

A total of 1012 people in the 117 households randomized to the plastic BSF-intervention villages and a total of 1031 people in the 143 households randomized to the control villages were compared. Shown in Table 1 and Table 2 are characteristics of the plastic BSF and control groups based on data collected during the initial cross-sectional questionnaire. When the two groups were compared based on the age and proportion of males and females in various age groups, the two groups differed (statistically) in the proportion of those that were <2, 2–4 and >4 years of age, although the proportions were similar. The proportion of males to females and the number of respondents that reported currently attending school were not significantly different between BSF and control households.

Plastic BSF and control group characteristics regarding water, sanitation, hygiene, and other household level characteristics are presented in Table 2. An overwhelming majority of households reported using surface water (collected from earthen dams, called “dugouts”) for drinking water in both dry and rainy seasons (71–98%). These dams or dugouts are typically shallow areas with slightly raised banks that capture rain or runoff water during the rainy season. This is the most common source for drinking water in this region of the country. Fewer control households (71%) compared to BSF households (94%) reported using surface water from earthen dams during the rainy season, a difference that was statistically significant. Households that reported using a source other than earthen dams during the rainy season reported using rainwater. 

**Table 1:** Age (as of May 2008), sex and education status of participants in control (no BSF) and intervention (BSF) households during a randomized controlled trial of the plastic BSF in rural Tamale, Ghana 2008.

**Individual Variables**	**Control**	**Intervention**	***p* values**
	(N = 1031)	(N = 1012)	Pearson χ^2^ test
**Age**	N (%)	N (%)	
Participants ≥ 5 years old	809 (78.5)	794 (78.5)	
Participants 2–4	146 (14.3)	116 (11.5)	
Participants <2	76 (7.4)	102 (10.2)	0.027 ^a^
**Sex**			
Male (≥5)	436 (42)	391 (39)	
Male (<5)	113 (11)	116 (11)	0.37 ^a^
Female (≥5)	373 (36)	401 (40)	
Female (<5)	108 (11)	104 (10)	0.12 ^a^
**Ever or currently attending school**	261 (25.3)	260 (25.7)	0.85

^a^ Pearson chi-squared test performed for proportions in all categories of age and gender comparing people in BSF and control groups.

**Table 2:** Selected characteristics regarding demographics, water and sanitation for control (no BSF) and intervention (BSF) households in a randomized controlled trial of the plastic BSF in rural Tamale, Ghana, June–December 2008.

Household Level Variables	Control	Intervention	*p* values
	(N = 143)	(N = 117)	(χ^2^ test)
Use surface water during dry season	95.0%	98.3%	0.16
Use surface water during rainy season	70.6%	94.0%	<0.001
Reported sieving drinking water through cloth	96.5%	96.6%	0.97
At least one person attending school in household	70.4%	74.8%	0.39
Lack access to sanitation ^a^	97.1%	98.3%	0.67
Multi-child household ^b^	56.7%	55.6%	0.86

^a^ Lack of access was characterized by the absence of any type of latrine or pour flush toilet. ^b^ Household has at least two children less than five years of age participating in the study.

Almost all households lacked access to any type of sanitation (97 and 98%, respectively for control and plastic BSF households). The two groups were not found to be significantly different when compared for other water and sanitation or demographic variables listed in Table 2, such as the practice of cloth sieving for water treatment, the proportion of households with at least one person currently attending school or households with more than one child less than five years of age. 

### 3.3. Diarrheal Disease

In order to examine the impact of the plastic BSF on diarrheal disease of participants, we compared the longitudinal prevalence between the two groups, plastic BSF intervention and control (no BSF) for the age groups of <2 years, all <5 years and all ages, both prior to the intervention and after installation of the plastic BSF as the intervention (Table 3). Before intervention, households that were randomly selected to receive plastic BSFs experienced slightly lower longitudinal prevalence of diarrheal disease than control households for all categories of age groups, a difference that was not statistically significant (adjusted LPR for all ages: 0.98, 95% CI: 0.23–3.94). During the plastic BSF intervention period, longitudinal prevalence of diarrheal disease of all age groups was significantly lower in the plastic BSF intervention group than in the control group. For example, for all ages, the BSF intervention group had 0.40 times the longitudinal prevalence of reported diarrhea as the control group (95% CI: 0.05, 0.80). This observation suggests significant protection from diarrheal disease by the plastic BSF during the four month intervention period.

**Table 3:** Adjusted longitudinal prevalence ratios for diarrheal disease, stratified by age, during the pre-intervention and intervention phases of a randomized controlled trial of the plastic BSF in rural Tamale, Ghana (2008).

Data collection Period	Age stratum	Unadjusted LP ^a^—Control Villages	Unadjusted LP ^a^—Plastic BSF Villages	Adjusted LPR (95% CI)
Baseline (May–August 2008)^ b ^	All	0.024	0.020	0.98 (0.23, 3.95) ^c^
	<2 years of age	0.081	0.10	1.56 (0.25, 9.83) ^d^
	<5 years of age	0.078	0.074	1.38 (0.19, 10.17) ^d^
Plastic BSF Intervention (September–December 2008)	All	0.012	0.0063	0.40 (0.05, 0.80) ^c^
	<2 years of age	0.028	0.015	0.37 (0.15, 0.90) ^d^
	<5 years of age	0.034	0.018	0.26 (0.07, 0.89) ^d^

^a^ LP—unadjusted longitudinal prevalence which was calculated as the total number of days with diarrheal disease over the total number of days observed; ^b^ Period of observation in villages prior to randomization and plastic BSF installation; ^c^ Longitudinal prevalence ratio and 95% confidence interval with plastic BSF as exposure adjusted for adjusted for categorical age of participant and clustering of diarrheal disease within household and villages; ^d^ Longitudinal prevalence ratio and 95% confidence interval with plastic BSF as exposure adjusted for clustering of diarrheal disease within household and villages.

When stratified by age group, the difference in longitudinal prevalence of diarrheal disease between participants in control and BSF households was even greater in children less than five years of age than in all age groups, with an adjusted LPR of 0.26, (95% CI: 0.07–0.89), corresponding to an estimated 74% reduction in diarrheal disease for plastic BSF participants compared to control participants. The level of diarrheal disease reduction for BSF participants compared to control participants was only slightly lower for children less than two years of age compared to all participants combined, with an (adjusted LPR of 0.37 (95% CI: 0.15, 0.90)), corresponding to an estimated 63% diarrheal disease reduction. 

### 3.4. Water Quality Analysis

Household drinking water quality was compared over the entire study period for plastic BSF and control households. The geometric mean concentrations of *E. coli* and mean turbidities of household drinking water for the baseline and intervention periods are compared in Table 4. Before the intervention, plastic BSF households and control households had water with very high geometric mean concentrations of *E. coli* and turbidity: 724 and 832 MPN *E. coli* /100 mL, respectively (control and plastic BSF). Likewise, households in both groups had drinking water with high geometric mean turbidities; 95 and 85 NTU for control and plastic BSF, respectively. Neither *E. coli* nor turbidity levels of household water were statistically significantly different between the two groups prior to the plastic BSF intervention (two sample t-test for turbidity (*p* = 0.23) and for *E. coli*, (*p* = 0.22)).

**Table 4:** Geometric mean *E. coli* concentrations and turbidities of household drinking waters for the control and plastic BSF groups before and after plastic BSF intervention in a randomized control trial in rural Tamale, Ghana (2008).

Baseline (May–August 2008)			Plastic BSF Intervention (September–December 2008)			
**Water Quality Parameter**	Control HH Water	Plastic BSF HH Water	Control HH Water	Plastic BSF Untreated Water^ b^	Plastic BSF Direct Filtrate	Plastic BSF Stored Filtrate
***E. coli *(95%CI) ^a^**	724 (631–851)	832 (724–977)	490 (426–549)	437 (380–501)	16 (13–20)	76 (62–91)
	*(N = 516)*	*(N = 424)*	*(N = 587)*	*(N = 385)*	*(N = 382)*	*(N = 381)*
**NTU**	95 (83–109)	85 (74–98)	25 (23–27)	47 (42–51)	15 (13–17)	15 (14–18)
	*(N = 523)*	*(N = 430)*	*(N = 787)*	*(N = 527)*	*(N = 524)*	*(N = 527)*
**% *E. coli* Reductions ^c^**	--	--	--	--	97	85
**% NTU Reductions ^c^**	--	--	--	--	67	66

^a^ Geometric mean and 95% confidence interval for *E. coli* (MPN/100mL) and turbidity of household (HH) drinking water; ^b^ Untreated water refers to water that was taken from households prior to any treatment. This is the assumed contamination level before the BSFs were used to treat the water.; ^c^ *E. coli* and NTU reduction calculated as log_10_ reduction = log_10_ influent – log_10_ effluent and then the log_10_ reductions were transformed into %.

After the plastic BSF intervention, households with the plastic BSF demonstrated appreciable improvements in drinking water quality compared to the control household without the BSF (Table 4). During the intervention period, *E. coli* concentrations were similar in the water that was collected and stored in the household before treatment (490 and 437 MPN/100mL for control and BSF groups, respectively; *p* value = 0.27). For the plastic BSF group, *E. coli* levels in water from the plastic BSF were significantly lower than those in the stored untreated water, (16 MPN/100mL and 437 MPN/100mL, in BSF direct filtrate and untreated stored water, respectively, *p* value < 0.0001). Plastic BSF treated and stored water was also significantly improved based on *E. coli* levels compared to untreated stored water in BSF households (76 MPN/100 mL and 437 MPN/100 mL, respectively; *p* < 0.0001). However, plastic BSF treated and stored water had significantly higher *E. coli* concentrations than water taken directly from the plastic BSF outlet, with 76 MPN/100 mL and 16 MPN/100 mL, respectively; *p* < 0.0001. After the plastic BSF was installed, both the control and plastic BSF intervention groups had collected stored water with lower turbidity as compared to the baseline phase (near 100 NTU prior to intervention and <50 NTU for both groups during the intervention phase). However, plastic BSF households had significantly lower turbidity for water treated by the plastic BSF (15 NTU compared to 27 NTU for control households). Unlike *E. coli* levels, turbidity levels of water did not change significantly during storage. 

Control and plastic BSF households were asked (at each household visit) whether or not they had performed treatment to their water. Prior to plastic BSF intervention, households reported sieving the water through a cloth prior to drinking for 97% of all observations. Boiling and chlorine were cited as treatment for 0.2% and 0.15% of the observations, respectively. During the intervention period, the control group reported high levels of cloth-sieving (93% of all observations) but no additional treatment. The plastic BSF group did not report any additional treatment beyond filtration with the plastic BSF during the intervention period.

In an attempt to measure adherence to the plastic BSF intervention, households reported the frequency of weekly plastic BSF use and use of the plastic BSF filtered water for drinking. During the weekly observations, none of the plastic BSF intervention households reported not using the BSF to filter water in the previous 7 days. In addition, in very few observations (3% all household observations in plastic BSF households) users report not drinking the plastic BSF filtered water. 

### 3.5. Plastic BSF Performance

The plastic BSF achieved a geometric mean 97% reduction of *E. coli* when untreated water in BSF households was compared to water direct from the BSF outlet. However, the *E. coli* reduction was much lower (85%) when comparing untreated water in BSF households to BSF-treated and stored water. A geometric mean reduction of 67% for turbidity was found comparing untreated water to water directly from the BSF; a similar reduction was found comparing untreated to BSF treated and stored water (66% geometric mean reduction). 

**Figure 3:**
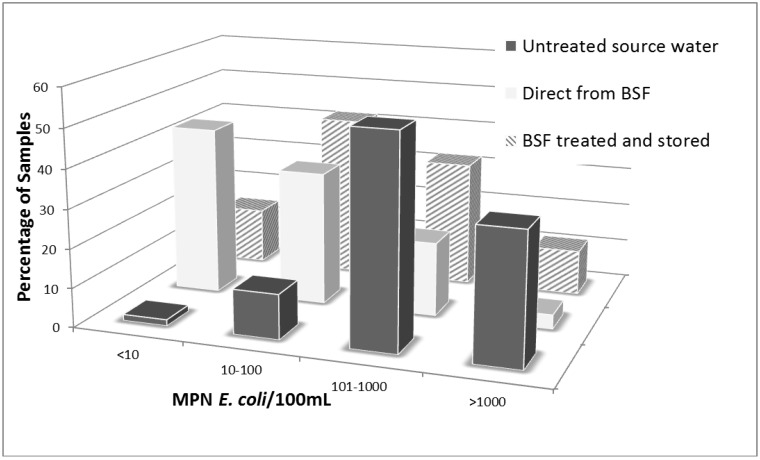
Comparison of categorical *E. coli* concentrations in different water samples from plastic BSF households during intervention period of the RCT of the plastic BSF in rural Tamale, Ghana (2008).

When compared on a categorical basis of order of magnitude concentration ranges as a basis for categorizing risk levels posed by the water, plastic BSF treated water had significantly fewer samples in high risk *E. coli* concentration categories as compared to untreated source water (*p* < 0.0001, Pearson’s chi-squared test). As shown in Figure 3, 44% and 15% of water samples taken directly from the plastic BSF outlet and the plastic BSF treated and stored water, respectively, had less than 10 MPN *E. coli*/100mL (considered low risk water) as compared to only 1.5% of water in BSF households prior to treatment. Furthermore, 77 and 56% of samples taken from the plastic BSF outlet directly or plastic BSF treated and stored, respectively, had fewer than 100 MPN *E. coli*/100mL (considered moderate risk) as compared to only 12% of all samples in this category prior to plastic BSF treatment in plastic BSF intervention households. Overall, there was significant improvement in categorical concentrations of *E. coli* for plastic BSF treated drinking water and plastic BSF treated and stored water compared to untreated water.

## 4. Discussion

To our knowledge, this is the first study to assess the ability of plastic BSF to reduce the longitudinal prevalence of diarrheal disease in Ghana. There are relatively few studies examining household water filtration in communities in this region of the world and in water sources as turbid as the ones found in this study. We documented a significant reduction (60%) in diarrheal disease for households who were asked to use a plastic BSF compared to control households that were given no BSF during the study period. One similar study, performed in Kenya with a concrete housing intermittently operated slow sand filter, found a 54% reduction in diarrhea prevalence [8]. They also found that the reduction of diarrheal disease was substantially higher (77%) when comparing households only using unimproved surface water sources for their drinking water supply [8]. Similarly high reductions (80%) in diarrheal disease were also reported in a randomized controlled trial of a ceramic water filter in South Africa and Zimbabwe [16]. The results from our study suggest reductions in diarrheal disease that are consistent with similarly designed trials on household water filters in other countries in this region [8,17]. 

The results from this study are also similar to the diarrheal disease reduction results we documented (as part of the three country trial of the plastic BSF) from an RCT in Cambodia, where diarrheal reductions in households using the plastic BSF were similar (59%) to those reported here during a five-month intervention trial [12]. In Honduras, the diarrheal disease reduction results of a plastic BSF RCT were similar in magnitude to those reported here although this was not statistically significant [13]. Overall, the results for diarrheal disease reduction in BSF households compared to control households observed in this study suggest greater or comparable reductions compared to those obtained from trials of concrete BSFs in other regions of the world such as Cambodia and the Dominican Republic, which demonstrated 47% [9], and 54% reductions in diarrhea illness risks [7], respectively, in children <5 years of age. These results also compare favorably to peer-reviewed studies of other HWT technologies such as chlorine disinfection, which has been found to achieve reductions in diarrheal disease of about an average 30% in many regions around the world [17].

Researchers, however, have recently questioned the validity of results of unblinded, randomized controlled trials lacking a placebo such as this one, suggesting that there may be a significant source of bias where households with the intervention may under-report diarrheal disease [18,19,20]. Because this is the first study of the plastic BSF in Northern Ghana, we did not include a placebo filter. Furthermore, due to the high turbidity of the surface water used as drinking water by households, it would have been difficult to blind participants to the treatment. Boisson *et al*. [21] recently performed a placebo controlled trial of a household filter in the Democratic Republic of Congo and experienced considerable challenges in designing and implementing a neutral filter as a placebo. We are unaware of any study that successfully employed a placebo filter at point-of-use in any developing country setting.

Additional research on the plastic BSF and other HWT technologies should attempt to measure health impacts in more objective ways that can help to eliminate bias. These may include incorporating diagnostic procedures to detect intestinal infections or including anthropometric measurements of children for longer-term health outcomes such as was performed in a recent trial of solar disinfection in Kenya [22]. However, even child anthropometry can be subject to measurement error and may not completely eliminate the question of bias [23,24].

### 4.1. Effect of the Plastic BSF on Household Drinking Water Quality

During the four months of intervention with the plastic BSF, we documented significant improvements in household water quality for both *E. coli* and turbidity of plastic BSF treated water. Because the lack of access to improved drinking water was high in these communities (71–98% used surface water which was collected from earthen dams) and the water accessed was highly microbiologically contaminated and often very turbid, this study provides important evidence regarding the potential application of the plastic BSF for locations where safe water access is limited. During the study, the plastic BSF demonstrated an average 97% reduction of *E. coli* in BSF-treated water, which is similar to reductions seen both in the laboratory and in the field for concrete BSFs and other filtration technologies [8,16,25,26,27]. For the untreated water of this study, when *E. coli* concentrations were ≥1000 MPN/100mL, the plastic BSF averaged 99% reduction (data not shown). As demonstrated in laboratory studies of a similarly designed plastic BSF, protozoan parasites and bacterial removals can be as high as ≥99% [27,28]. Virus removals may not be as robust (<90%) and may be more dependent on the conditions of the biological activity and the biofilm in the BSF [29]. Under the conditions examined during our study, we expect bacterial and protozoan pathogens to be effectively removed by the plastic BSF at levels similar to the removals of the bacterial indicators (97% or more). Furthermore, there is potential for virus removals greater than what has been documented in the laboratory due to the potential for virus attachment to particles in the turbid raw water and the likelihood of robust biofilms as the result of this highly turbid raw water. Rotavirus have been documented as an important pathogen in the region [30] and further study on the potential for this BSF to remove viruses under the conditions of this study is warranted.

Although there were significant reductions of *E. coli* in household drinking water samples as a result of treatment with the plastic BSF, there was also evidence of recontamination during storage of plastic BSF-treated water (Figure 3, Table 4). This type of recontamination has been documented in previous studies of the concrete BSF [7,10,31]. As shown in Figure 1, although the water may leave the outlet tube of the plastic BSF relatively uncontaminated, during storage of treated water there are ample opportunities for bacterial contamination to be re-introduced via hands, dippers and even the storage container itself. The opportunity for bacterial recontamination has been documented for other treatment options that do not provide a residual disinfectant, such as boiling [32]. For these types of technologies, additional training regarding safe and hygienic storage of treated water should be included to reduce bacterial recontamination. 

While the turbidity reduction in household drinking water by the plastic BSF in the intervention group was significant compared to untreated water and to control household water turbidity, the average turbidity of treated drinking water of 14 NTU was still higher than the WHO suggested limit of 5 NTU. Few studies have examined treatment of water in regions where the main source of drinking water is highly turbid surface water. Crump *et al.* [33,34] demonstrated a significant reduction in diarrheal disease, bacterial contamination and turbidity for households using a combined flocculant-disinfectant in Kenya, with turbidities reduced from >100 NTU to <5 NTU in 50–70% of water samples treated. Mwabi *et al.* [35] reported that a range of locally produced point-of-use water filters, including BSFs, consistently reduced the turbidities of surface waters with an average turbidity of about 40 NTU to <5 NTU in South Africa. Also important to consider was that turbidity decreased for both control and plastic BSF households during the intervention period. This may be due to water quality improvements at the point of collection, possibly associated with changes in rainfall. 

Limitations of this study include randomization at the village level which resulted in a small number of clusters, having participants unblinded to the exposure and lack of a placebo. Additional limitations include use of self-reported diarrhea, with a 7-day recall of diarrhea, as the health outcome measure and the relatively short duration of the intervention. However, such limitations are common in other studies of HWT technologies such as the ceramic water filter and chlorination. The short duration and frequent observations of this study are important limitations of its design because evidence suggests that many point-of-use household water interventions show decreased performance effectiveness over time [19]. A recent study investigated the impact of household survey on health behaviors and found that the act of survey itself may impact respondents’ behaviors [36]. Therefore, studies of longer duration and those that are designed to eliminate household survey impact on respondents’ behaviors should also be considered for the BSF in future studies. Design and conduct of studies that evaluate implementation programs extending over longer periods of time would also be more informative of the longer term impacts of the plastic BSF on correct and consistent daily use, water quality and health. 

Despite the aforementioned limitations in this study, the results can still be compared to other rigorous and widely cited studies of HWT technologies and their impact on the diarrheal disease risks of the users. In particular, these results confirm those of past RCTs of the concrete BSF by documenting that the plastic BSF has the ability to reduce the longitudinal prevalence of diarrheal disease and significantly improve drinking water quality, even in a cohort of households that is primarily using unimproved, turbid and microbiologically contaminated surface water from earthen dams for their main source of drinking water.

## 5. Conclusions

To our knowledge, this is only one of two known health impact studies on the performance of the BSF in Sub-Saharan Africa and is the first on the plastic BSF on this continent. Positive results were found for the ability of the plastic BSF filter to reduce diarrheal disease and improve water quality in communities of the Northern Region of Ghana using highly turbid and microbiologically contaminated surface water collected from earthen dams as their drinking water source. However, more research is necessary to document plastic BSF performance, continued and effective use and overall sustainability after installation and in the absence of intensive sampling or other monitoring. 
