# Correction: Stauber, C.E., *et al.* Evaluation of the Impact of the Plastic BioSand Filter on Health and Drinking Water Quality in rural Tamale, Ghana. *Int. J. Environ. Res. Public Health* 2012*,* *9,* 3806–3823

**DOI:** 10.3390/ijerph110909154

**Published:** 2014-09-03

**Authors:** Christine E. Stauber, Byron Kominek, Kaida R. Liang, Mumuni K. Osman, Mark D. Sobsey

**Affiliations:** 1 School of Public Health, Georgia State University, P.O. Box 3995, Atlanta, GA 30302, USA; 2 Department of Environmental Sciences and Engineering, Gillings School of Global Public Health, University of North Carolina-Chapel Hill, Campus Box 7431, Chapel Hill, NC 27599, USA; E-Mails: byron.kominek@gmail.com (B.K.); kliang@email.unc.edu (K.R.L.); mark_sobsey@unc.edu (M.D.S.); 3 Cowater International Inc., NORST, P.O. Box 1476, Tamale, Ghana; E-Mail: hoiinahii@gmail.com

The authors wish to make the following amendments to their paper published in *International Journal of Environmental Research and Public Health* [1]:

1. Page 3806, line 5 in Abstract: the sentence “During the study, the longitudinal prevalence ratio for diarrhea comparing households that received the plastic BSF to households that did not receive it was 0.40 (95% confidence interval: 0.05, 0.80), suggesting an overall diarrheal disease reduction of 60%.” should read “During the study, the longitudinal prevalence ratio for diarrhea comparing households that received the plastic BSF to households that did not receive it was 0.41 (95% confidence interval: 0.18, 0.92), suggesting an overall diarrheal disease reduction of 59%.”
2. Page 3813, the last sentence “Before intervention, households that were randomly selected to receive plastic BSFs experienced slightly lower longitudinal prevalence of diarrheal disease than control households for all categories of age groups, a difference that was not statistically significant (adjusted LPR for all ages: 0.98, 95% CI: 0.23–3.94).” should read “Before intervention, households that were randomly selected to receive plastic BSFs experienced slightly lower longitudinal prevalence of diarrheal disease than control households for all categories of age groups, a difference that was not statistically significant (adjusted LPR for all ages: 0.94, 95% CI: 0.22–4.08).”
3. Page 3814, line 5 in the first paragraph, the sentence that reads “For example, for all ages, the BSF intervention group had 0.40 times the longitudinal prevalence of reported diarrhea as the control group (95% CI: 0.05, 0.80).” should read “For example, for all ages, the BSF intervention group had 0.41 times the longitudinal prevalence of reported diarrhea as the control group (95% CI: 0.18, 0.92).”
4. Page 3814, Table 3, the items under “Adjusted LPR” corresponding to the corrections above have been revised. The correct Table 3 should be:

**Table 3:** Adjusted longitudinal prevalence ratios for diarrheal disease, stratified by age, during the pre-intervention and intervention phases of a randomized controlled trial of the plastic BSF in rural Tamale, Ghana (2008).

Data Collection Period	Age Stratum	Unadjusted LP ^a^—Control Villages	Unadjusted LP ^a^—Plastic BSF Villages	Adjusted LPR (95% CI)
Baseline (May–August 2008)^ b ^	All	0.024	0.020	0.94 (0.22, 4.08) ^c^
	<2 years of age	0.081	0.10	1.48 (0.24, 9.27) ^d^
	<5 years of age	0.078	0.074	1.38 (0.19, 10.17) ^d^
Plastic BSF Intervention (September–December 2008)	All	0.012	0.0063	0.41 (0.18, 0.92) ^c^
	<2 years of age	0.028	0.015	0.37 (0.15, 0.90) ^d^
	<5 years of age	0.034	0.018	0.26 (0.07, 0.89) ^d^

^a^ LP—unadjusted longitudinal prevalence which was calculated as the total number of days with diarrheal disease over the total number of days observed; ^b^ Period of observation in villages prior to randomization and plastic BSF installation; ^c^ Longitudinal prevalence ratio and 95% confidence interval with plastic BSF as exposure adjusted for adjusted for categorical age of participant and clustering of diarrheal disease within household and villages; ^d^ Longitudinal prevalence ratio and 95% confidence interval with plastic BSF as exposure adjusted for clustering of diarrheal disease within household and villages.

The authors would like to apologize for any inconvenience caused to readers by these changes.
